# A phenomenological description of BslA assemblies across multiple length scales

**DOI:** 10.1098/rsta.2015.0131

**Published:** 2016-07-28

**Authors:** Ryan J. Morris, Keith M. Bromley, Nicola Stanley-Wall, Cait E. MacPhee

**Affiliations:** 1 School of Physics and Astronomy, University of Edinburgh, James Clerk Maxwell Building, Peter Guthrie Tait Road, Edinburgh EH9 3FD, UK; 2 Division of Molecular Microbiology, School of Life Sciences, University of Dundee, Dundee DD1 5EH, UK

**Keywords:** BslA, emulsions, interfacial science, interfacial protein

## Abstract

Intrinsically interfacially active proteins have garnered considerable interest recently owing to their potential use in a range of materials applications. Notably, the fungal hydrophobins are known to form robust and well-organized surface layers with high mechanical strength. Recently, it was shown that the bacterial biofilm protein BslA also forms highly elastic surface layers at interfaces. Here we describe several self-assembled structures formed by BslA, both at interfaces and in bulk solution, over a range of length scales spanning from nanometres to millimetres. First, we observe transiently stable and highly elongated air bubbles formed in agitated BslA samples. We study their behaviour in a range of solution conditions and hypothesize that their dissipation is a consequence of the slow adsorption kinetics of BslA to an air–water interface. Second, we describe elongated tubules formed by BslA interfacial films when shear stresses are applied in both a Langmuir trough and a rheometer. These structures bear a striking resemblance, although much larger in scale, to the elongated air bubbles formed during agitation. Taken together, this knowledge will better inform the conditions and applications of how BslA can be used in the stabilization of multi-phase materials.

This article is part of the themed issue ‘Soft interfacial materials: from fundamentals to formulation’.

## 1. Introduction

Many bacteria live within communities known as biofilms [1,2]. A biofilm typically consists of an extracellular polymeric matrix composed of polysaccharides, proteins and DNA which gives mechanical stability to the community [1–4]. The bacterium *Bacillus subtilis* lives within a biofilm that is notable for its highly wrinkled morphology and extremely hydrophobic surface [5]. The hydrophobicity of the biofilm surface has been primarily attributed to the surface active protein BslA [6–8]. The interfacial activity of BslA is achieved via its amphiphilic structure: a large ‘cap’ of hydrophobic residues is conjoined to a hydrophilic immunoglobulin-like domain [9].

This structural configuration is reminiscent of a family of unrelated surface active proteins known as the fungal hydrophobins [10–12]. Hydrophobins also possess a large surface-exposed patch that is stabilized by a network of disulfide bonds [13–15]. The highly amphiphilic nature of their structure causes them to spontaneously partition to a hydrophobic interface. Upon adsorption, hydrophobins self-assemble into an elastic surface layer with corresponding elastic moduli greater than most other interfacially bound protein surface layers [16,17]. Hydrophobins have garnered significant interest for their potential applications as surface modifiers [18–24] and stabilizers for emulsions and foams [16,25–29].

While BslA superficially appears to be a ‘bacterial hydrophobin’, its mechanism for interfacial activity is distinct from the fungal hydrophobins. BslA lacks the network of disulfide bonds that rigidifies the structure of hydrophobins. Notably, under normal solution conditions, BslA does not aggregate and remains highly soluble. BslA achieves this through the structural flexibility of the hydrophobic cap. While in an aqueous environment, the hydrophobic amino acids that constitute the cap orient themselves so as to minimize their interaction with the surrounding environment. Once the protein reaches an interface, the cap undergoes a structural transition, inserting the hydrophobic residues into the non-polar phase and forming a highly ordered elastic film [9,30].

In this work, we describe several structures formed by the stabilization of an interface by BslA. First, we study the formation of BslA-stabilized air bubbles in aqueous solutions formed by agitation or shaking. Owing to the mechanical strength of BslA elastic films, the shape of these microbubbles is highly aspherical with large aspect ratios. We investigate their properties under a range of solution conditions and monitor their stability. Second, we observe the formation of ‘flakes’ that are generated during stirring or shearing of a BslA aqueous solution. These flakes precipitate from the interface and remain for extended periods of time in the aqueous environment. We find that the structure of these film ‘flakes’ contains crystalline-like domains very reminiscent of those we have reported previously [30]. Third, we find that under compressive or shear forces BslA elastic films form macroscopic, tubular formations. We study these structures via optical and electron microscopy in addition to rheo-imaging methods. Taken together, this work highlights several structural morphologies of BslA over a wide range of length scales.

## 2. Experimental section

### (a) Protein production and purification

BslA was expressed and purified using standard protocols—see the electronic supplementary material for a complete description.

### (b) Turbidity measurements of air bubbles

For measuring the turbidity of BslA solutions as a function of concentration, a serial dilution was prepared from the stock protein solution. The pH of solutions was adjusted using 1 M HCl and 1 M NaOH. The pH for each solution was read using a Mettler–Toledo FiveEasy pH meter. A 100 μl sample of each solution was placed into polymerase chain reaction tube strips and capped. The samples were then vigorously manually shaken for 60 s. The agitated solutions were immediately dispensed into a 96-well plate using a multi-pipette and the turbidity measured in a BMG Fluostar Omega plate reader at 600 nm. The time between the end of agitation and the first read was approximately 30 s. Measurements were repeated three times and were highly reproducible.

### (c) Optical microscopy of air bubbles

BslA samples were manually shaken for 60 s and immediately pipetted onto a cavity well slide. A glass coverslip was then placed over the sample and immediately imaged in bright-field mode using an Olympus BX50 microscope and a QImaging QICAM Fast 1394 CCD camera. Movies were captured of the dissipation of the bubbles and analysed using ImageJ software.

### (d) Langmuir trough compressions and microscopy

A KSV-Nima minitrough was used in these experiments. The trough and barriers were thoroughly cleaned using distilled, deionized water and ethanol. MilliQ water (resistivity 18 MΩcm) was added to the trough and a paper Wilhelmy plate placed within this subphase and allowed to equilibrate. The surface was then aspirated to remove any impurities. Compressions were performed until the surface pressure did not change by more than 0.3 mN m^−1^. A 200 μl sample of 1.5 mg ml^−1^ BslA was gently placed onto the surface of the subphase using a microsyringe. The sample was spread with the barriers at a fully open position. Compressions were performed at a rate of 
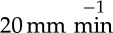
. Microscopy images were obtained in bright-field mode using a microscope set-up that was built in-house (Nikon objectives).

### (e) Scanning electron microscopy of BslA film tubules

Samples were prepared by compressing a BslA film in the Langmuir trough. After the compression was complete, 200 μl of 10% glutaraldehyde was added to the aqueous phase. The film was allowed to cross-link for 2 h. The cross-linked film was then lifted onto a scanning electron microscope (SEM) stub from below. Samples were allowed to dry and then sputter-coated with platinum. Experiments were performed on a Hitachi 4700 II cold field emission scanning electron microscope.

### (f) Transmission electron microscopy

BslA-stabilized interfacial ‘flakes’ were prepared by first shearing a solution of BslA (0.1 mg ml^−1^) in a rotorstator (IKA Ultra Turrax T10) at level 6 (30 000 r.p.m.). The resultant stabilized air bubbles creamed, and the subphase was replaced with phosphate buffer (25 mM, pH 7). The creamed samples were left at 4°C overnight until an insoluble sediment was observed. A 5 μl droplet containing a small amount of sediment was pipetted onto carbon-coated copper grids (Cu-grid; TAAB Laboratories Equipment Ltd) and allowed to dry. The grid was then stained with 5 μ*l* of 2% uranyl acetate for 5 min before being wicked from the side with filter paper. Images were acquired using a Philips/FEI CM120 BioTwin transmission electron microscope.

### (g) Analysis of wrinkle lifetime of pendant drops

A 0.025 mg ml^−1^ solution of BslA was expelled from a syringe (1.83 mm needle diameter) into glycerol trioctanoate to a volume of 40 μl. The drop volume was controlled via Krüss EasyDrop software. After a designated equilibration time, a volume of 3 μl was retracted. The subsequent wrinkles in the BslA film were captured via a CCD camera at 2 fps. After formation, the wrinkles were monitored over a period of 10 min. The videos of the recordings were split into individual frames, and image analysis was performed on each frame. We selected a line profile across the neck of the droplet, where wrinkles are most apparent, and plotted the greyscale values (from 0 to 255) of each pixel along this line using ImageJ. To plot the relaxation profiles, the greyscale value of the pixels was normalized to values between 0 and 1 and background corrected. A total of six wrinkles per droplet were analysed. The value reported is the average of these six wrinkles; errors bars are standard deviation.

## 3. Results and discussion

### (a) Air bubbles stabilized by BslA

#### (i) Turbidity measurements

**Figure 1.**
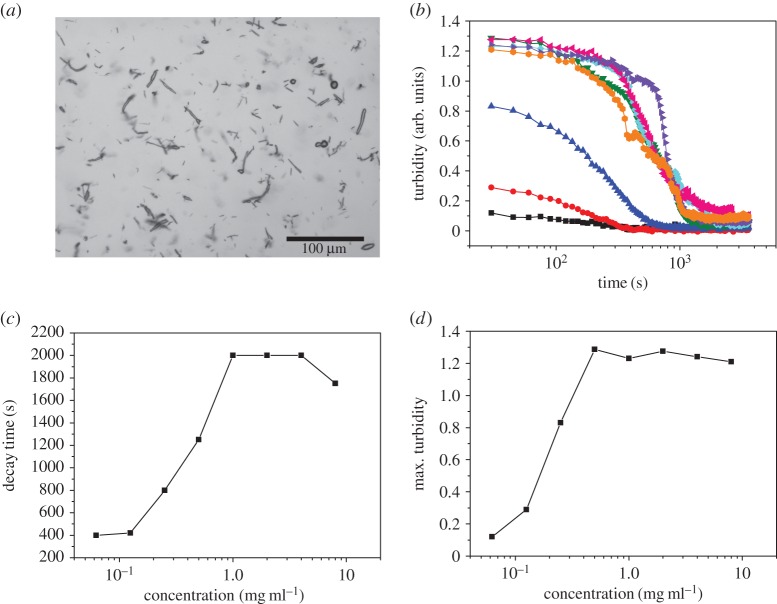
(*a*) Image taken of a sample shows the stabilization of air bubbles in a 1 mg ml^−1^ BslA solution. Irregularly shaped and elongated air bubbles are the predominant morphology. (*b*) Turbidity as a function of bulk BslA concentration. Black squares (0.0625 mg ml^−1^), red circles (0.125 mg ml^−1^), blue upside triangles (0.25 mg ml^−1^), green downside triangles (0.5 mg ml^−1^), cyan diamonds (1 mg ml^−1^), pink left triangles (2 mg ml^−1^), purple right triangles (4 mg ml^−1^) and orange octagon (8 mg ml^−1^). (*c*) The decay time (the time at which the decay curves in (*b*) reach a baseline turbidity) shows a concentration dependence up to 1 mg ml^−1^. Above this concentration, the decay times become constant. (*d*) A similar trend is observed for the value of maximum turbidity.

We observed that when solutions of BslA were agitated or shaken the previously clear samples became notably turbid. After a length of time, the turbidity would dissipate and ultimately the samples cleared. Optical microscopy (figure 1*a*) revealed that the sample contained highly aspherical bubbles with high aspect ratios (8.7±5.1; see figure S1 in the electronic supplementary material for the distribution of aspect ratios). Similar irregularly shaped air bubbles stabilized by the hydrophobin HFBII have also been observed [31]. BslA-stabilized air bubbles differ from those stabilized by HFBII in that the turbidity of HFBII-stabilized samples remains indefinitely unless sonicated. The decay of BslA-stabilized air bubbles is also in contrast to oil-in-water emulsions stabilized by BslA which remain stable for time scales of the order of years (see electronic supplementary material, figure S1).

In order to better understand the process of air bubble dissipation in BslA solutions, we first measured the turbidity of BslA samples as a function of protein concentration (figure 1*b*). We observe that for BslA concentrations greater than 0.5 mg ml^−1^ the turbidity decay curves superimpose. Below this value there is a clear relationship between the protein concentration, the amount of bubbles formed, and their rate of decay. Here we define the decay time as the time for the decay curve to reach a baseline turbidity. Figure 1*c* shows clearly that for concentrations greater than or equal to 1 mg ml^−1^ the decay time reaches a constant value. Below this concentration, there is a clear concentration dependence on the decay time. A similar trend is also observed for the value of maximum turbidity (figure 1*d*). These results indicate that, for the experimental protocols used, at BslA concentrations over 0.5 mg ml^−1^, there is excess protein available to coat the amount of interface created by vigorous shaking. Below this concentration, the amount of BslA limits the area of interface that can be stabilized. One could potentially ‘separate’ the decay curves by putting more energy into the system (by more vigorous shaking or for longer), whereby more bubbles would be created in samples above 0.5 mg ml^−1^, which in turn would take longer to decay; however, under the conditions used here, the amount of interface generated becomes limiting.

**Figure 2.**
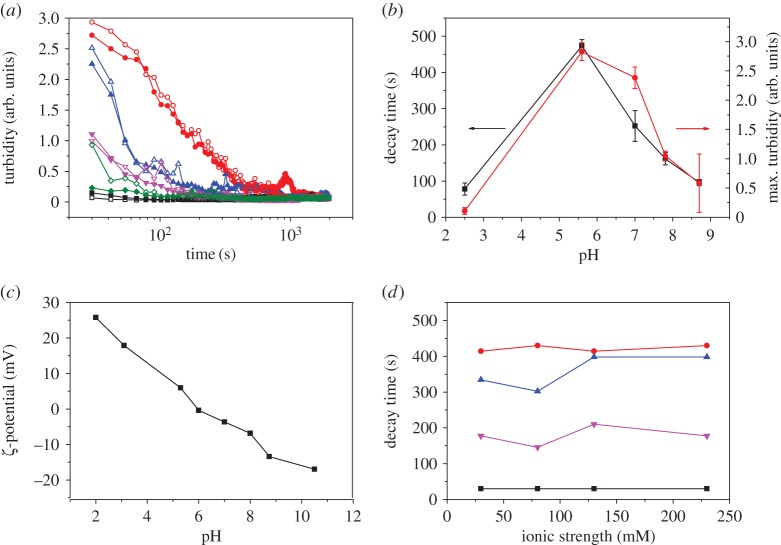
(*a*) Turbidity decay as a function of pH: black squares (pH 2.5), red circles (pH 5.6), blue triangles (pH 7), pink downside triangles (pH 7.8) and green diamonds (pH 8.7). The closed and open symbols represent two separate experiments. (*b*) Plot of decay time (black squares) and maximum turbidity (red circles) as a function of pH. We found a peak in both observables at approximately pH 5.5. (*c*) ζ-potential of BslA-stabilized emulsion (20% decane in water) droplets as a function of pH. (*d*) Plot of decay times as a function of ionic strength for pH 2 (black squares), pH 5.5 (red circles), pH 7 (blue triangles) and pH 8.8 (pink downside triangles). We found a very weak dependence on ionic strength on these observables.

We then studied the dissipation of BslA-stabilized bubbles under different solution conditions. First, we measured the turbidity decay as a function of pH. Figure 2*a* shows the decay curves obtained from these experiments. What is immediately notable is that pH has a strong influence on the formation and stability of the air bubbles. Figure 2*b* plots both decay times and maximum turbidity and shows that there is a peak in both observables at pH approximately 5.5. There is little bubble stabilization at acidic and basic pHs. Monomeric BslA in solution has an isoelectric point calculated to be 9.2. Therefore, it is curious that so little bubble stabilization occurs at basic pH (7–9). We ascribe this behaviour to a shift in the pI of BslA when bound to the air–water interface. Indeed, the pI of HFBII was found to shift when adsorbed to an air bubble [31]. This pI change for adsorbed HFBII is thought to arise from the fact that a large proportion of the protein is no longer in contact with water, therefore only a fraction of the functional groups are ionizable. A shift in pI was also observed for emulsion films stabilized by β-lactoglobulin [32,33]. In this case, the shift was attributed to conformational changes that occur upon surface adsorption.

In order to test this hypothesis, we measured the ζ-potential of emulsion droplets stabilized by BslA. We chose to measure emulsion droplets because they are extremely long lived thus avoiding the problem of air bubble dissipation over time. The result can be found in figure 2*c*. We found that the isoelectric point of the emulsion droplets occurs at approximately pH 5.5. This result is in agreement with the observations above.

The addition of salt had little influence on the formation and stability of bubbles formed over a range of pH values. We added increasing concentrations of NaCl to phosphate buffer at several pH values and the resulting turbidity decay curves were measured. It is clear from figure 2*d*, which shows the decay time as a function of ionic strength, that ionic strength has little effect on the characteristics of the bubbles. Indeed, the decay times are relatively constant and similar to the values found in figure 2*b*.

#### (ii) Monitoring the dissipation of air bubbles using optical microscopy

**Figure 3.**
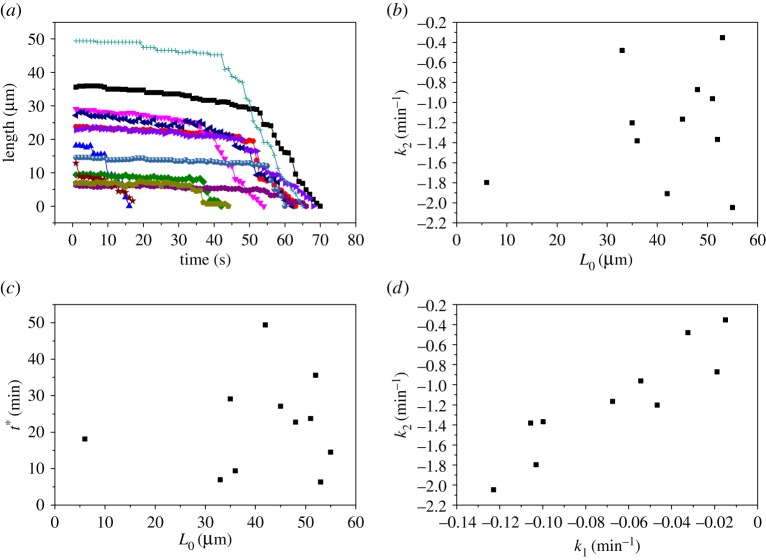
Results from analysis of video microscopy on the dissipation of air bubbles stabilized by BslA. (*a*) There are characteristic decay curves for all bubbles measured. There is an initial slow decay regime characterized by a decay rate *k*_1_. At a transition time t*, the bubbles undergo a rapid decay phase that is described by a second decay rate, *k*_2_. Each colour represents a measurement of an individual bubble. (*b*,*c*) There is no correlation between the rapid decay rate, *k*_2_, and the initial length of the bubble, *L*_0_, or the transition time, t*. (*d*) The decay rates are correlated, however, showing that those bubbles that begin a quicker decay in the first phase also have a concomitant rapid decay in the second. The protein concentration used was 0.1 mg ml^−1^ at pH 7.

Here we wish to understand the mode in which the bubbles dissipate. Video microscopy was performed on the turbid BslA samples, and the resulting decay of the bubbles was measured by determining the contour length of each bubble as a function of time (aspect ratios and length distributions of these elongated bubbles can be found in the electronic supplementary material, figures S2 and S3). As can be seen in figure 3*a*, there is a clear characteristic shape to the decay curves, which is composed of two phases. First, there is a slow decay regime which we characterize by a rate *k*_1_. After a length of time, t*, a sudden rapid decay of each bubble occurs, which is reflected in the rate *k*_2_. We found that there is no correlation between the fast decay rate, *k*_2_, and the initial length of the bubble, *L*_0_, or the transition time, t* (figure 3*b*,*d*). However, we also found that a smaller *k*_1_ correlates with a smaller *k*_2_ (figure 3*d*). In other words, the quicker the decay in the first phase, the faster it is in the second phase. The decay curves found from these optical experiments, where we have measured individual bubbles, are in agreement with the decay curves found through the measure of the bulk turbidity of the solution, especially at higher BslA concentrations (figure 1*b*). Indeed, at high protein concentrations, there is a clear slow decay phase followed by a rapid decay, just as was found in these optical experiments.

Bubble decay occurs via contraction, i.e. they decrease in size via retraction of the elongated tips, rather than any decrease in the bubble width. This shrinkage occurring from the poles of the bubbles is likely to be a curvature-induced phenomenon, because the greatest stresses on the BslA film occur at these locations. Such stresses may cause defects in the order or arrangement of the individual BslA molecules in the interfacial film. High aspect ratio liquid droplets have been shown to undergo a similar shape change to BslA-stabilized air bubbles [34,35]. In that case, rod-like liquid droplets, containing a soft wax interior, could be induced to collapse back to a spherical shape by heating or dilution. The contraction of the aspherical droplets was attributed to a simple force balance between the higher Laplace pressures at either end of the cylinder against the elasticity of the interior network. Such a simple model could also potentially explain our observations. There is, however, a notable difference. Our system is effectively the inverse of that described in [34,35], since in the BslA-stabilized example the elastic network is on the exterior. With the presence of an exterior elastic film, it raises a question whether the idea of interfacial tension, and in turn, a Laplace pressure, is applicable.

**Figure 4.**
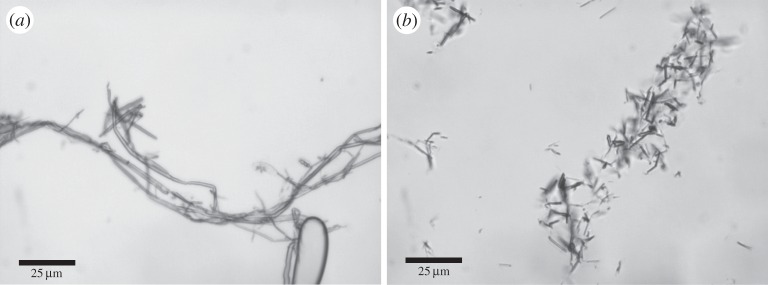
Air bubbles created in a 1 mg ml^−1^ solution of BslA are chemically cross-linked using glutaraldehyde. (*a*) The typical elongated morphology is intact. (*b*) Large aggregates of bubbles are commonly observed owing to the cross-linking process. However, these cross-linked bubbles do not dissipate and remain in solution indefinitely.

Finally, we note that the dissipation of the bubbles may be arrested by covalently cross-linking the interfacial BslA layer using glutaraldehyde. As can be seen in figure 4*a*,*b*, the bubbles remain in their typical elongated morphology. Owing to the cross-linking process, however, aggregation of the bubbles frequently occurs (figure 4*b*). The fact that these bubbles no longer disappear lends further credence to the notion that structural defects in the interfacial film could be the underlying cause of this effect. Substantially cross-linking the surface layer can effectively ‘patch over’ any structural defects locking the structure in place.

An overall question remains: why are elongated air bubbles coated in BslA unstable while oil in water emulsions are stable? The answer probably lies in both the kinetic and thermodynamic properties of BslA interfacial adsorption. We have previously shown [30] that BslA has to overcome an energy barrier in order to adsorb to an interface. Combined with the fact that an air–water interface is less hydrophobic than an oil–water one, there may not be a sufficient kinetic window for BslA to adsorb and form a robust and well-organized interfacial layer on the surface of an air bubble.

**Figure 5.**
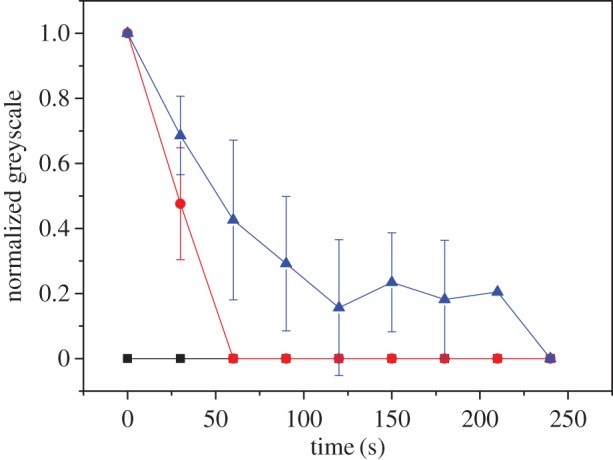
Relaxation of BslA films as a function of equilibration time: 2 min (black squares); 5 min (red circles); 10 min (blue triangles). Films were produced in a pendant drop from a 0.03 mg ml^−1^ solution of BslA. Wrinkles were monitored and the average lifetimes of six wrinkles are reported (error bars are standard deviation).

To reinforce this point, we monitored the relaxation of a BslA film formed on a pendant drop after compression at an oil–water interface as a function of equilibration time. We observe that there is a clear dependence between the equilibration time and the robustness of the film (figure 5). Taken together, we propose that the dissipation of air bubbles arises from non-optimal packing of BslA units at the poles owing to higher curvature in these regions, in conjunction with the slow kinetics of formation of the cohesive, elastic surface layer.

### (b) Structures formed from BslA elastic films

#### (i) Structured BslA film ‘flakes’ produced by agitation

**Figure 6.**
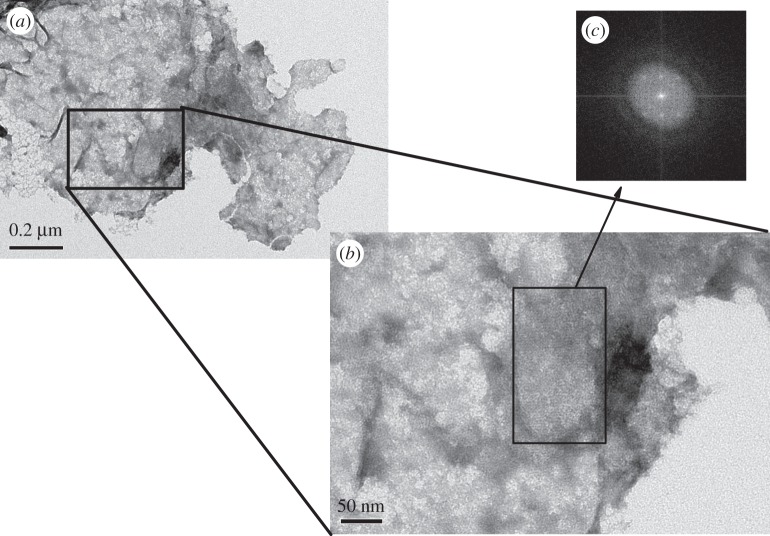
TEM of BslA film ‘flakes’. When agitating a BslA solution (0.5 mg ml^−1^, pH 7), a sediment appears after some time. This sediment is not aggregated material but ‘flakes’ of BslA films. (*a*) A representative sample of a flake as imaged by TEM. (*b*) A FFT was performed on the region indicated by the black rectangle and shows that the organization of BslA within the flake is crystalline-like (*c*).

When agitating a BslA solution via vortexing or other methods, a particulate sediment appears. This sediment remains in solution over a time scale of weeks before slowly disappearing. The sediment appears as ‘flakes’ of stabilized interface when viewed by transmission electron microscopy (TEM). A representative image is found in figure 6*a*. Notably, the constituents of the flake possessed lattice-like organization. Figure 6*b* shows a region of the flake that was well stained. Performing a fast Fourier transform (FFT) of this region (figure 6*c*) shows clear peaks, indicating crystalline-like organization. Very similar crystalline-like domains were observed for BslA films formed directly on a TEM grid substrate [30]. This result indicates that the crystalline domains found via deposition of BslA on the substrate are not an artefact of this deposition process but are intrinsic to the organization of BslA at an interface.

Looking back at figure 1*b*, the baseline of the decay curves for BslA-stabilized bubbles does not return to zero. Upon inspection, a sediment is found in the bottom of the container similar to the ‘flake’ sediment seen by simple vortexing. Therefore, we attribute this non-zero baseline value to the sedimentation of BslA flakes formed from the decaying air bubbles.

#### (ii) Compression of BslA films in a Langmuir trough creates macroscopic tubular structures and wrinkled sheets

**Figure 7.**
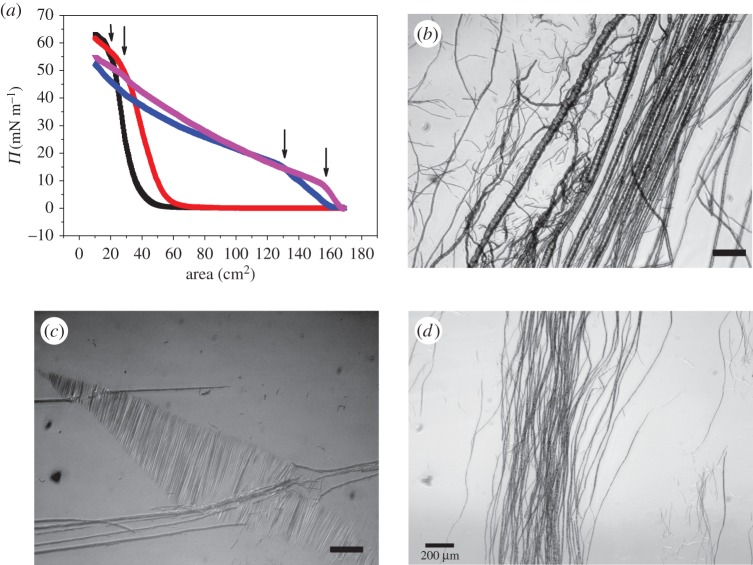
(*a*) Surface pressure/area isotherms for BslA films at different equilibration times. Black, 0.5 h; red, 1 h; blue, 5 h; and pink, 16 h. Arrows show the turnover in isotherms, which is indicative of layer collapse. (*b*–*d*) Examples of tubular structures found in the buckling zones after compression in the Langmuir trough. For reference, the barriers would be parallel to the left/right edge of each image. The quantity of BslA added to the trough at the start of the experiment was 254 μg.

To investigate the properties of a BslA film at an air–water interface further, we performed experiments in a Langmuir trough where BslA was applied to a MilliQ water subphase. In these experiments, BslA was applied to the surface of the water phase and allowed to equilibrate for varying lengths of time. In Langmuir trough experiments, the surface pressure is measured; this is the change in surface tension owing to the addition of the surfactant (*Π*=*γ*_0_−*γ*, where *γ*_0_ is the surface tension of the bare interface). Resulting *Π*-area isotherms are shown in figure 7*a* for several different equilibration times. We found that, for increasing equilibration times, the change in *Π* occurs at larger and larger areas. This is due to the fact that BslA, while applied directly to the interface, is soluble in water and some of the protein will be lost to the subphase upon application. The larger areas at which the change in *Π* occurs are simply caused by the protein that was initially lost to the bulk during application diffusing back to the interface. This is one reason why trying to obtain a mean molecular area measurement for a soluble surfactant is not reliable. For the 1 h equilibration isotherm, we note that, when *Π*≈55 mN m^−1^, there is a turnover in the isotherm which indicates the point of film buckling or failure. We observed wrinkles or deformations on the surface of the film at this turnover point in the isotherm. Observationally, the buckling occurs in parallel and close to the barriers. Furthermore, we found that, with increasing equilibration times, the surface pressure at the buckling instability decreases. Therefore, the failure pressure depends upon the amount of protein at the interface. A very similar result is found for HFBII under compression in a Langmuir trough [36]. For colloidal systems and particle rafts, such a dependence on the quantity of material at the interface for the buckling pressure was posited to be caused by a two-dimensional Janssen-like effect where the granular effects of force propagation play a critical role [37]. However, in the HFBII system, it was shown that such a granular model is not entirely applicable to a hydrophobin layer and that the film could be considered more as an elastic sheet [36].

After a compression was completed, we observed a clear band of deformed film located near to each barrier. These deformations sometimes spanned nearly the entire width of the trough, i.e. of the order of tens of millimetres. We observed that these deformations would disappear after ≈12 h.

We investigated these deformation zones in more detail by using an optical microscope arranged over the Langmuir trough. Microscopy revealed that these deformation zones typically consisted of two morphologies: large tubular-like structures (figure 7*b*–*d*) and wrinkled sheets (figure 7*b*). The tubular structures, on average, were aligned parallel with the barriers. It is noteworthy that these tubules are structurally reminiscent of the elongated stabilized air bubbles that were discussed in the previous section. Figure 7*c* shows tubules appearing to lie on top of, and disturbing, the film wrinkles. This implies that the tubules are separate entities from the underlying elastic film, i.e. the tubules are lying on top of the film.

In addition to observing tubule structures, we also found wrinkled zones in the elastic film. One example of this can be seen in figure 7*c*. For an elastic membrane, there exist three possible tension regimes when it is placed under a stretching or compressional stress: positive, negative or tension-free. For a negative tension state, it is energetically favourable for the membrane to bend or buckle out of plane, forming wrinkles [38,39]. The wavelength of the wrinkles is determined by the mechanical properties of the membrane, namely the bending elasticity, *k*_*c*_. It was shown [39] that, by measuring the wrinkle wavelength, one may obtain a value for the bending rigidity,
3.1
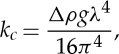
where Δ*ρ* is the difference in mass densities of the two phases on either side of the interface and *g* is the acceleration owing to gravity. Moreover, the sheet tension, *σ*_*m*_, may be found from the value of *k*_*c*_ given by
3.2
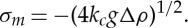


This analysis is applicable in the linear regime, that is, when the out-of-plane deformations are small. In this case, *σ*_*m*_ and the wavelength are solely determined by *k*_*c*_ and the effect of gravity, resulting in a sinusoidal membrane profile. The wavelength of wrinkles is then independent of *L*, the distance between the barriers, and Δ*L*, the decrease in distance between the barriers upon compression. The degree of compression (Δ*L*/*L*) only controls the amplitude of the deformation. However, the analysis can be extended to the nonlinear regime when the out-of-plane deformations are not small. In this case, one obtains ‘toothed’, non-harmonic membrane profiles. For these non-harmonic cases, a value of *k*_*c*_ cannot be obtained from the expression above.

We analysed several images of wrinkled BslA films (distinct from tubule structures) from Langmuir trough compressions and obtained the wavelength, λ. We found a distribution of wavelengths (see figure S4 in the electronic supplementary material) with a mean value 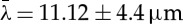. Moreover, the amplitude does not seem to be constant throughout the entire wrinkle zones. Both these observations raise questions as to whether these deformations are within the linear regime. In the nonlinear regime, one can observe multiple wavelengths λ_1_ and λ_2_, where λ_1_ is superimposed on a much longer λ_2_ [39,40]. In our case, we do not find such a bimodal distribution.

Earlier, we found that the tubule structures were aligned parallel with the barriers of the Langmuir trough. In contrast, the wrinkle zones are often not parallel and one may find wrinkle zones close to each other, but oriented at differing angles. It has been observed that BslA forms highly organized domains with neighbouring domains oriented at varying angles [30]. Therefore, this observation may be explained by the fact that the BslA film is not a single contiguous layer but consists of domains with varying orientational order. In this case, the propagation of stress would not be homogeneous and could result in deformations such as those that we observed.

With these caveats in place, let us assume that the deformations we observed are within the linear regime, so that we can obtain an estimate of the bending elasticity from the expression in equation (3.1) using the mean value of λ. This gives a value of the bending elasticity *k*_*c*_=9.9×10^−20^ J and a membrane tension *σ*_*m*_=−6.2×10^−5^ mN m^−1^. These values are quite close to those obtained for HFBII: *k*_*c*_=1.1×10^−19^ J and *σ*_*m*_=−6.6×10^−5^ mN m^−1^ [31]. So BslA films have similar structural properties to HFBII, at least within contiguous domains.

It should be noted that such a low negative value of the membrane tension is in disagreement with the buckling surface pressure we observed in the Langmuir trough compressions (figure 7*a*). In that case, the onset of buckling always occurs at positive tension values. One explanation is that the method for measuring interfacial tension using contact methods (such as a Wilhelmy plate) in the presence of an elastic layer is not valid.

As pointed out for HFBII, such a large bending elasticity and small membrane tension could have important implications for the application of BslA in foams. The large elasticity could provide mechanical stability to the foam, whereas the low tension would translate into slow Ostwald ripening owing to low Laplace pressure differences across bubble interfaces.

**Figure 8.**
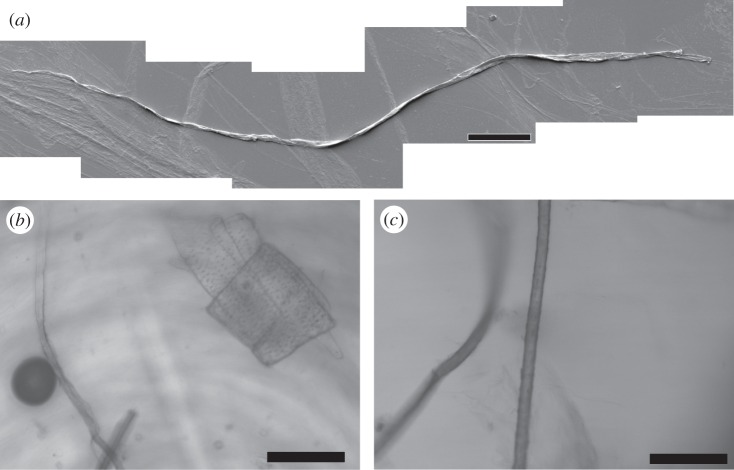
(*a*) SEM image of a tubule obtained from a glutaraldehyde cross-linked sample lifted from a Langmuir trough. The quantity of BslA initially applied to the trough was 254 μg. The tubule appears to be a rolled piece of film with a hollow interior. Scale bar, 50 μm. (*b*,*c*) Examples of tubular structures formed under shear in a cone and plate rheometer. An initial concentration of 0.8 mg ml^−1^ was used for these experiments. Scale bar, 100 μ*m*.

To further image the tubule structures, we chemically cross-linked the film using glutaraldehyde. The cross-linked structures were then lifted onto an SEM stub for imaging. Figure 8*a* shows a tubule imaged using the SEM. From this image, it appears that the tubule consists of a rolled and hollow piece of film.

Finally, we imaged BslA solutions under shear in a cone and plate rheo-imaging set-up [41]. We found that under these conditions very similar tubule structures are formed at the interface (figure 8*b*,*c*). Indeed, a large number of different structures were observed from tubular structures to free sheets (figure 8*b*). What is apparent is that all of these structures emanate from the interface and are not generated from within the bulk of the solution. When the frame rate of the camera is synchronized with the oscillation frequency, we clearly observe that these structures migrate from the interface into the bulk.

## 4. Conclusion

In this work, we have given a phenomenological description of several self-assembled structures made from the interfacial protein BslA which spans length scales from nanometres to millimetres. First, we have demonstrated that BslA can form transiently stable air bubbles upon agitation of BslA solutions. We propose that rapidly formed bubbles dissipate over time owing to structural defects in the ordered surface layer. Second, macroscopic tubular structures, highly reminiscent of the smaller air bubbles found in solution, are formed upon shear stresses applied in a Langmuir trough compression or a cone and plate rheometer. Like the hydrophobins, BslA is an attractive candidate for applications concerning surface modification and stabilization of multi-phase materials. This work begins to highlight several important aspects of properties of BslA films and solution-state behaviour that can play an important role when considering applications and conditions for the utilization of BslA in new materials.

## Supplementary Material

Supplementary Information: A Phenomenological Description of BslA Assemblies Across Multiple Length Scales

## Data accessibility

The data in this paper have been deposited in the University of Edinburgh DataShare repository and may be found at http://dx.doi.org/10.7488/ds/1355.
