# Supplementary material for: A phenomenological description of BslA assemblies across multiple length scales
Source: Philos Trans A Math Phys Eng Sci. 2016 Jul 28;374(2072):20150131. doi: 10.1098/rsta.2015.0131 (PMC4920280; doi:10.1098/rsta.2015.0131)
Supplement: Supplementary Information: A Phenomenological Description of BslA Assemblies Across Multiple Length Scales [file rsta20150131supp1.pdf]

Phil. Trans. R. Soc. B
doi: 10.1098/not yet assigned

Supplementary Information: A Phenomenological Description of BslA Assemblies Across Multiple Length Scales

Ryan J. Morris[1], **Keith M. Bromley**[1], **Nicola Stanley-Wall**[2] **and Cait E. MacPhee**[1,*]

[1]School of Physics & Astronomy, University of Edinburgh
[2]Division of Molecular Microbiology, College of Life Sciences, University of Dundee
[*]To whom correspondence should be addressed: School of Physics & Astronomy, University of Edinburgh, James Clerk Maxwell Building, Peter Guthrie Tait Road, Edinburgh, EH9 3FD, Tel: +44 (0) 131 651 7067, Fax: +44 (0) 131 650 5902, Email: cait.macphee@ed.ac.uk

Experimental Section

Protein Expression & Purification: The BslA proteins used in this study were purified using an adaptation of the method described in previous work. The plasmids encoding overexpression constructs containing the BslA42-181 coding region fused to GST and separated by a tobacco etch virus (TEV) protease recognition site (wild-type bslA: pNW1128 and bslAL77K: pNW1189) were transformed into *E. coli* BL21 (DE3) pLysS. The transformed cells were grown in LB broth (supplemented with ampicillin (100 μg.mL^{-1})) overnight, then inoculated into autoinduction media (again supplemented with ampicillin (100 μg.mL^{-1})) at a ratio of 1:1000 (vol:vol). The cultures were incubated at 37°C with shaking at 150 rpm until they reached an optical density at 600 nm of 0.9, then the temperature was reduced to 18°C overnight. Centrifugation at 4000 g for 45 min was used to collect the cells, which were then frozen at - 80°C until needed. The cells collected from 1 litre of culture were resuspended in 25 mL of purification buffer (50 mM HEPES, 250 mM NaCl, pH 7.5) supplemented with Complete EDTA-Free Protease Inhibitors (Roche), before being lysed using an Emulsiflex cell disruptor (Avestin) with pressure at 15000 psi applied three times to each sample. Insoluble cell debris and unlysed cells were removed by centrifugation at 27000 g for 20 min. 0.75 mL of Glutathione Sepharose 4B (GE Healthcare) beads was add to the cleared lysate from 1 L of cells and placed at 4°C with gentle agitation for 4 h to allow binding of the GST-tagged BslA protein to the beads. The beads and cell lysate was then loaded onto a single-use gravity flow column (Biorad) and the liquid allowed to flow through. The beads were then washed twice by the addition of 25 mL of the purification buffer to remove any unbound proteins. The washed beads were then collected and added to 25 mL of purification buffer supplemented with 1 mM DTT and 0.5 mg of purified TEV protease, and placed at 4°C overnight with gentle agitation. The solution containing the beads, released BslA, TEV protease and unbound GST was again loaded onto the gravity flow columns and the flow-through collected. To the flow-through 0.75 mL of fresh Glutathione sepharose beads and 0.25 mL of Ni- NTA (nickel-nitriloacetic acid) agarose beads (Qiagen) were added and the solution incubated at 4°C with gentle agitation overnight to remove the TEV protease and any unbound GST. The mixture was again passed through the gravity flow column and the purified protein collected in the flow-through. The protein was then concentrated using Vivaspin concentrators (Sartorius).

The proteins were then further purified by size-exclusion chromatography using a Superdex 75 10/300 GL column (GE Healthcare); the fractions containing pure BslA were combined and concentrated again using Vivaspin concentrators. When buffer exchange into 25mM phosphate buffer pH 7 was required it was done during the concentration step.

Phil. Trans. R. Soc. B
doi: 10.1098/not yet assigned

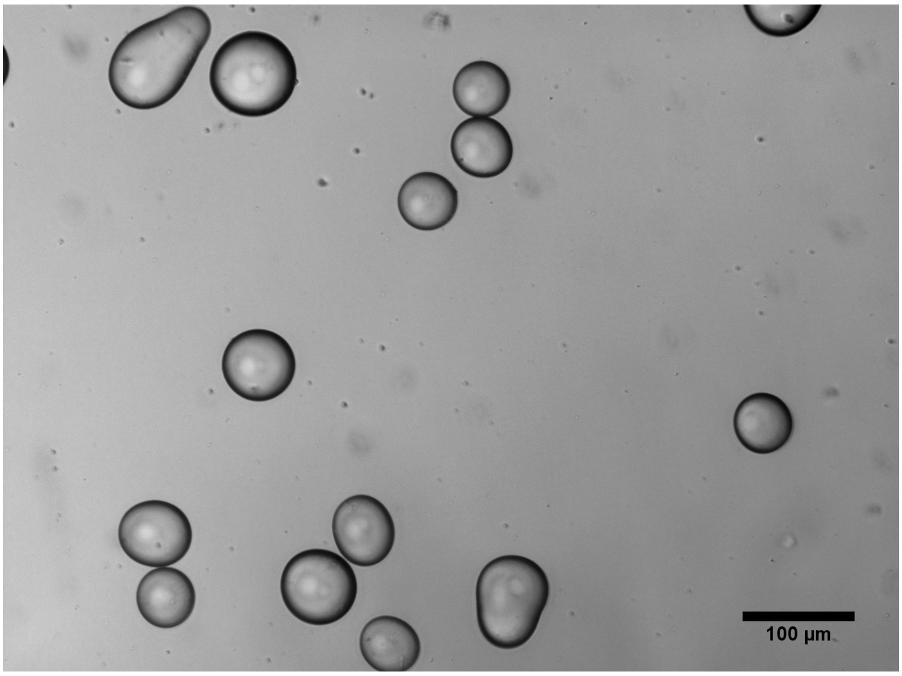

Figure S1: 20% decane in water emulsion stabilized by BslA. Typical emulsions show aspherical droplets. Lifetimes of emulsions are on order of years.

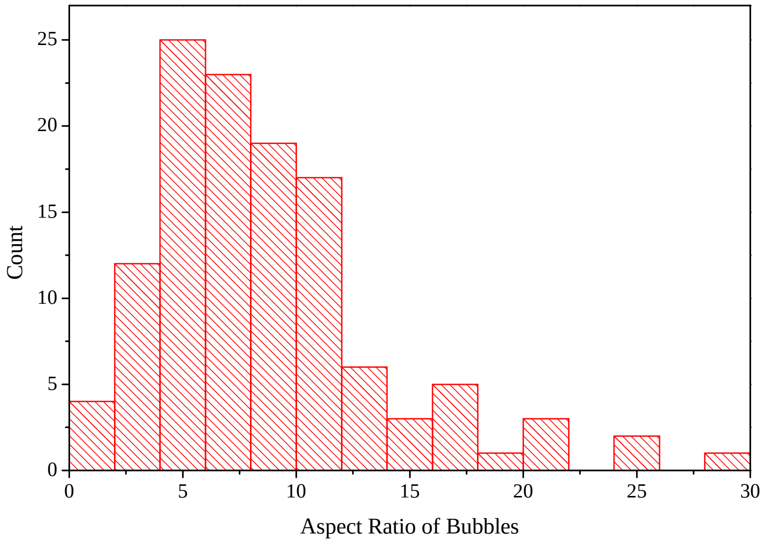

Figure S2: Aspect ratio distribution of bubbles obtained from optical microscopy.

doi: 10.1098/not yet assigned

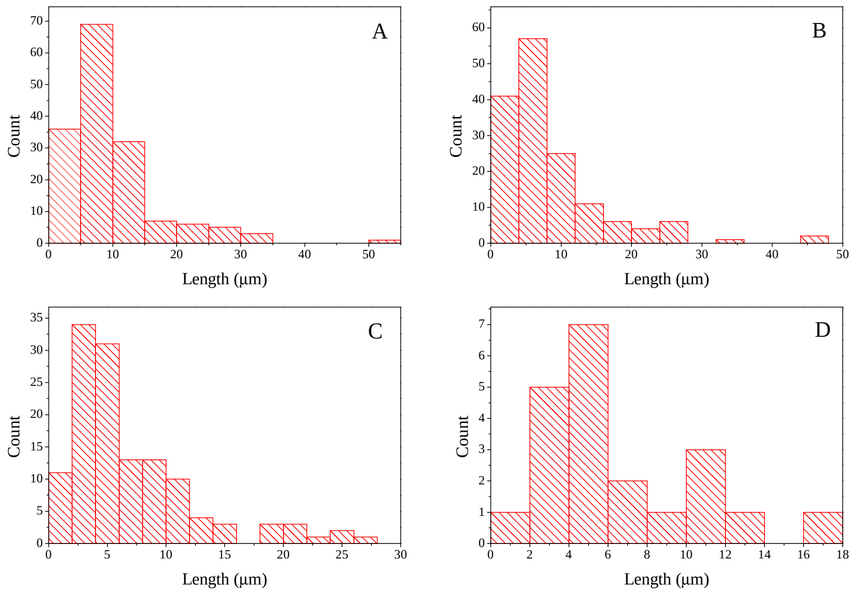

Figure S3: Length distribution of bubbles obtained from optical microscopy after (A) 0 sec, (B) 30 sec, (C) 50 sec, and (D) 70 sec.

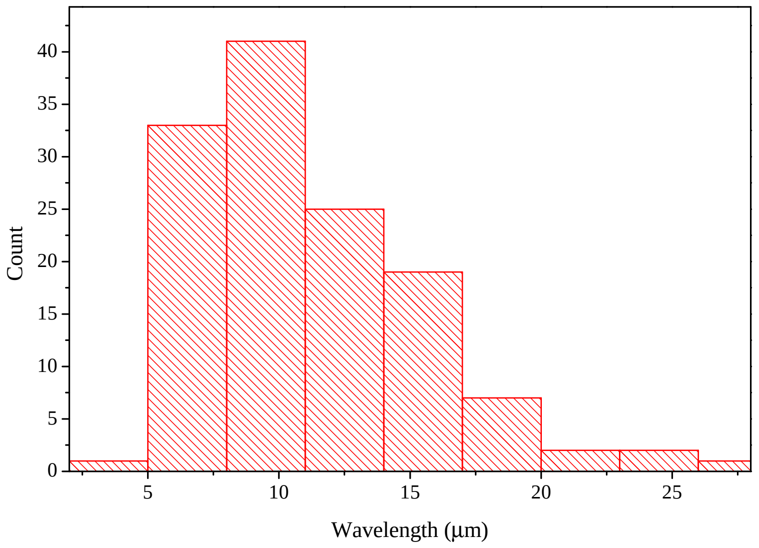

Figure S4: Distribution of wavelengths obtained from analysis of images of wrinkled films in Langmuir trough. We find the mean wavelength $\overline{\lambda} = 11.12 \pm 4.4$ μm; N=131.
